# Draft Genome Sequences of Two Commensal Enterococcus faecalis Strains Isolated from American Black Vultures (Coragyps atratus) in Brazil

**DOI:** 10.1128/mra.00057-22

**Published:** 2022-07-11

**Authors:** Andréa de Andrade Rangel Freitas, Stephanie da Silva Rodrigues Souza, Adriana Rocha Faria, Paul J. Planet, Vânia Lúcia Carreira Merquior, Lúcia Martins Teixeira

**Affiliations:** a Instituto de Microbiologia Paulo de Góes, Universidade Federal do Rio de Janeiro, Rio de Janeiro, Rio de Janeiro, Brazil; b Faculdade de Ciências Médicas, Universidade do Estado do Rio de Janeiro, Rio de Janeiro, Rio de Janeiro, Brazil; c Faculdade de Medicina, Universidade Federal do Rio de Janeiro, Rio de Janeiro, Rio de Janeiro, Brazil; d Perelman School of Medicine, University of Pennsylvania, Philadelphia, Pennsylvania, USA; e Pediatric Infectious Disease Division, Children’s Hospital of Philadelphia, Philadelphia, Pennsylvania, USA; f Sackler Institute for Comparative Genomics, American Museum of Natural History, New York, New York, USA; Loyola University Chicago

## Abstract

We report the draft genome sequences of two commensal Enterococcus faecalis strains (designated Ca-2 and Ca-18) recovered from the cloacae of two healthy American black vultures (Coragyps atratus) in Rio de Janeiro, Brazil. The strains were found to carry a variety of antimicrobial resistance and virulence-associated genes.

## ANNOUNCEMENT

Enterococcus faecalis is an opportunistic bacterium found in the intestinal microbiome of humans and animals (1, 2). The emergence of this species as a major nosocomial pathogen has been associated with its ability to acquire antimicrobial resistance and virulence genes (1, 3) and constitutes a significant concern for both human and veterinary medicine (1, 4, 5).

We present the draft genome sequences of two E. faecalis strains (Ca-2 and Ca-18) isolated from two American black vultures (Coragyps atratus) admitted to a wildlife rehabilitation center in Rio de Janeiro, Brazil. Cloacal content samples were inoculated into Enterococcosel broth (BD Microbiology Systems, USA). After 24 h at 37°C, an aliquot of each culture was streaked onto an Enterococcosel agar plate and incubated under the same conditions.

Genomic DNA was obtained from 1.5 mL of each culture grown in tryptic soy broth overnight at 37°C, using a Wizard genomic DNA purification kit (Promega, Madison, WI, USA); it was prepped using the Nextera XT kit and sequenced on a HiSeq 2500 sequencer (Illumina Inc., San Diego, CA, USA) with 125-bp paired-end reads. The read quality metrics were evaluated using FastQC v0.11.18 (6). The reads were trimmed (Trim Galore v0.6.5) (7), assembled (Unicycler v0.4.8) (8), and annotated (RASTtk v1.073) (9) using the resources of the Pathosystems Resource Integration Center (PATRIC) v3.6.9 (10). Default parameters were used for all software tools. Sequencing and assembly data for the two genomes are summarized in Table 1.

**TABLE 1:** Basic characteristics of the whole-genome assemblies of two Enterococcus faecalis strains isolated from American black vultures in Rio de Janeiro, Brazil

Parameter	Data for Enterococcus faecalis strain:	
	Ca-2	Ca-18
Genome size (bp)	3,059,826	3,072,615
No. of raw reads	490,885	624,609
GC content (%)	37.30	37.37
No. of contigs	59	92
Mean coverage (×)	46	58
No. of CDSs^*a*^	3,058	3,069
*N*_50_ (bp)	173,562	134,282
No. of tRNAs	52	53
No. of rRNAs	3	3
SRA accession no.	SRR8163627	SRR8163626
GenBank assembly no.	RJJO00000000.2	RJJP00000000.2

a CDSs, coding DNA sequences.

VirulenceFinder v2.0 (11) was used to identify genes playing important roles in the pathogenesis of enterococcal infections. Both strains harbored genes coding for gelatinase production (*gelE*), endocarditis antigen (*efaA*), aggregation substance (*agg*), and sortase (*srtA*), which are associated with adherence, aggregation, and invasion of host tissues (2, 4). Other virulence-associated genes identified in both strains included *cad*, *cOB1*, *cCF10*, and *camE* (coding for sex pheromones) (4, 12), *tpx* (protection against oxidative stress) (4, 13), and *elrA* (the enterococcal Rgg-like regulator gene associated with macrophage evasion) (4, 14). Genes associated with cytolysin production (*cylA*, *cylB*, *cylL*, and *cylM*) (2, 4) and collagen adhesion (*ace*) (4) were identified only in strain Ca-18.

ResFinder v4.1 (15) was used to identify the following major antimicrobial resistance genes in strain Ca-2: *ant(6)-Ia*, *aph(3′)-III*, *sat-4*, and *str* (aminoglycoside); *erm*(B) (macrolide, lincosamide and streptogramin B); *lnu*(G) (lincosamide); *tet*(L) (tetracycline); *cat*A (chloramphenicol); and *dfr* (trimethoprim). Both strains carried the *tet*(M) (tetracycline resistance) and *Isa*A (streptogramin resistance) genes. Mutations in GyrA (Glu88Gly) and ParC (Ser85Ile), implicated in fluoroquinolone resistance (16, 17), were identified in strain Ca-2 by alignment with reference sequences (GenPept accession numbers BAB69479.1 and BAB69481.1, respectively). Both strains contained the plasmid repUS43, and strain Ca-2 also carried rep_9c_ (18). One confirmed CRISPR region was identified in Ca-18 (19). Moreover, using PHASTER (20), one intact prophage was predicted in the Ca-2 genome and two intact prophages in the Ca-18 genome. Multilocus sequence typing (21) identified the strains as sequence type 33 (ST330) (Ca-2) and ST82 (Ca-18).

### Data availability.

The raw sequence reads have been deposited at the NCBI Sequence Read Archive (SRA) under the BioProject accession number PRJNA503970. The whole-genome shotgun project versions described in this paper are listed in Table 1.
